# Tensorial neutron tomography of three-dimensional magnetic vector fields in bulk materials

**DOI:** 10.1038/s41467-018-06593-4

**Published:** 2018-10-02

**Authors:** A. Hilger, I. Manke, N. Kardjilov, M. Osenberg, H. Markötter, J. Banhart

**Affiliations:** 1 Helmholtz Centre Berlin for Materials and Energy (HZB), Institute of Applied Materials, Hahn-Meitner-Platz 1, 14109 Berlin, Germany; 2 Department of Materials Science and Technology, Technische Universität Berlin, Hardenbergstraße 36, 10623 Berlin, Germany

## Abstract

Knowing the distribution of a magnetic field in bulk materials is important for understanding basic phenomena and developing functional magnetic materials. Microscopic imaging techniques employing X-rays, light, electrons, or scanning probe methods have been used to quantify magnetic fields within planar thin magnetic films in 2D or magnetic vector fields within comparable thin volumes in 3D. Some years ago, neutron imaging has been demonstrated to be a unique tool to detect magnetic fields and magnetic domain structures within bulk materials. Here, we show how arbitrary magnetic vector fields within bulk materials can be visualized and quantified in 3D using a set of nine spin-polarized neutron imaging measurements and a novel tensorial multiplicative algebraic reconstruction technique (TMART). We first verify the method by measuring the known magnetic field of an electric coil and then investigate the unknown trapped magnetic flux within the type-I superconductor lead.

## Abstract (web summary)

Mapping the distribution of magnetic fields inside bulk materials is challenging but crucial to understand and develop functional magnetic materials. Here the authors demonstrate the capability to visualize 3D vector magnetic fields inside materials using spin-polarized neutron tomography and tensorial reconstruction techniques.

## Introduction

Surveying magnetic fields inside solid matter is a difficult challenge but new methods are developing that allow one to visualize and quantify magnetic structures: Techniques for surface measurements such as magneto-optical Kerr microscopy^1^, electron magnetic exchange force microscopy^2,3^ and spin-polarized scanning tunneling microscopy^3^ on the one hand, or transmission techniques such as soft X-ray holography/dichroism microscopy^4–6^ and Lorentz transmission electron microscopy/holography^7–9^ on the other have been used to probe the internal magnetic structure of thin samples (up to some 100 nm thickness) even in three dimensions^10^.

However, there is still a fundamental gap: The complete 3D structure of a magnetic vector field in (thick) bulk samples is inaccessible by any of these techniques. A first approach in this direction provided maps of the magnetic domains within a thin film rolled into a cylinder^11^. More recently, an approach based on ptychographic X-ray nano-tomography has been demonstrated to provide magnetic contrast^12,13^. This technique has been further extended to investigate the magnetic vector field in 3D in a GdCo_2_ sample^14^. The contrast used is based on the X-ray magnetic circular dichroism (XMCD) at X-ray absorption edges of the corresponding material and restricts the usable X-ray energies to that of the X-ray absorption edges of the corresponding materials, which affects the maximum achievable penetration depths.

The fundamental problem of imaging and measuring magnetic vector fields inside solid matter is the necessity to use a probe that is sensitive to magnetic fields on the one hand, but penetrative enough to reach the region of interest inside a material on the other. Light and X-rays interact mainly with atomic electron shells and only weakly with a magnetic field directly. Due to their spin, electrons are very sensitive to magnetic fields, but they have only limited penetration depths. In contrast, neutrons satisfy both conditions as their spin is affected by magnetic fields due to their intrinsic magnetic moment while they penetrate deeply into many materials, especially metals, due to their zero electric charge and weak interaction with the atomic shell. It has been shown that neutron phase grating interferometry^15,16^ can be used to investigate magnetic domains^17^ and other magnetic structures such as vortex-lattice domains^18^ two-dimensionally and three-dimensionally^19^ in bulk samples, but only the domain walls could be visualized and no information on the magnetic field direction was obtained. On the other hand, spin-polarized neutron imaging provides access to the magnetic field direction due to the precession of neutrons when passing a magnetic field and the measurable change of the spin-polarization direction of a beam. Several approaches have been suggested to make use of the potential of spin-polarized neutron imaging for 3D magnetic vector field measurements in bulk materials^20–23^, but quantitative tomography has only been demonstrated for simple, one-dimensional magnetic fields, where the field vectors vary only in strength but not in direction^24,25^.

Here we show a way to overcome the current limits by designing a neutron imaging setup equipped with four spin-flippers and two spin-polarizers and obtaining a set of nine individual tomography measurements instead of just one. The data obtained serve as an input for a specially developed reconstruction algorithm used to extract the full three-dimensional magnetic vector field distribution.

## Results

### Basic principles

**Fig. 1:**
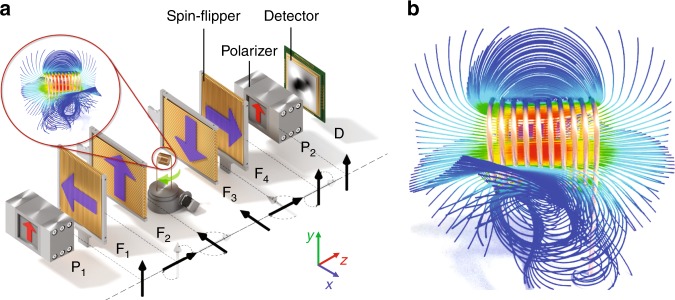
Tensor tomography. **a** Schematic drawing of the setup used for tensor tomography with spin-polarized neutrons, comprising spin polarizers (P), spin flippers (F) and a detector (D). **b** Selected magnetic field lines around an electric coil (calculation, see text and Methods)

A sketch of the imaging setup is shown in Fig. 1. After monochromatization the neutrons pass the first spin polarizer P_1_. In a magnetic field a neutron spin can take only one of two possible eigenstates, namely spin up |↑〉 or spin down |↓〉. The spin-polarizer shown in Fig. 1 absorbs neutrons with spin down |↓〉 so that only spin up |↑〉 neutrons can pass. Ignoring for a moment the flippers (F_1_ to F_4_) the now spin-polarized neutron beam, intensity profile *I*_0_(*x*, *y*), passes through the sample, during which in each ray of the beam the spin orientation may change depending on the magnetic vector field distribution along the ray. Finally, another spin polarizer P_2_ called the spin analyzer and a 2D detector are used to measure the transmitted intensity *I*(*x*, *y*) given by:

1 $$I\left( {x,y} \right) = I_0\left( {x,y} \right) \cdot \underbrace {\exp \left( { - {\int}_{{\mathrm{path}}} {\mu _{{\mathrm{att}}}} \left( s \right){\mathrm d}s} \right)}_{\leq 1({\mathrm{attenuation}}\,{\mathrm{by}}\,{\mathrm{sample}})} \cdot \frac{1}{2}\underbrace {\left[ {1 + {\mathrm{cos}}\left( {\vartheta \left( {x,y} \right)} \right)} \right]}_{\leq 1({\mathrm{spin}}\,{\mathrm{rotation}})},$$

where *μ*_att_(*s*) is the linear attenuation coefficient in the sample along path *s* and defines the first term. The second term occurs because neutrons pass the polarization analyzer P_2_ with an angle *ϑ*(*x*, *y*) between the beam polarization vector ${\vec{\mathrm P}}$ and the ${\vec{\mathrm y}}$ axis (see coordinate system in Fig. 1) at location (*x*, *y*). Therefore, from the measurement of transmission *I*(*x*,*y*) alone, only the polar angle between ${\vec{\mathrm P}}$ and ${\vec{\mathrm y}}$ is obtained. The azimuthal angle remains unknown and requires additional measurements using two pairs of spin flippers F_1_-F_4_.

The first pair of spin-flippers F_1_/F_2_ is used to rotate the initial spin polarization vector of the neutron beam (parallel to ${\vec{\mathrm y}}$ after polarization by P_1_) into one of three possible axes ${\vec{\mathrm x}}$, ${\vec{\mathrm y}}$, and ${\vec{\mathrm z}}$ by an angle of $\frac{{\mathrm{\pi }}}{2}$. After passing the sample, the spin-polarization of the neutrons can be in any direction. A second pair of spin-flippers F_3_/F_4_ (see Fig. 1a) is used in combination with the spin analyzer to probe the spin polarization changes of the neutron beam in any of the three directions ${\vec{\mathrm x}}$, ${\vec{\mathrm y}}$, and ${\vec{\mathrm z}}$ separately by applying $\frac{{\mathrm{\pi }}}{2}$ spin flips again. Altogether 3 × 3 different measurements are performed for different combinations of neutron beam spin polarization before and after the sample. In this way, all components of the magnetic field in the sample can be probed. Further details are explained in Methods.

Beside the difficulty to precisely set-up and adjust the various neutron-optical elements, a further major challenge is to find a mathematical reconstruction procedure that allows one to extract information from the nine 3D data sets and eventually obtain three sets of 3D data representing each magnetic vector field component. Here, we develop a technique that resembles the well-known, although due its complexity barely used, multiplicative algebraic reconstruction technique (MART) algorithm that was only made for scalar values, but can be extended to use tensors, then called tensorial MART (TMART), see Methods.

### Magnetic vector field of an electric coil

We apply a three-stage procedure to verify and apply the new method: First, the new TMART technique is tested by letting it reconstruct a simulated tomography experiment on a calculated magnetic field of an electric coil. Second, a real coil producing a known field is probed by neutrons and TMART applied. Comparison with the all-simulated experiment validates the experimental approach. Finally, an unknown complex magnetic field inside a real sample is measured. Accompanying simulations based on simplified current patterns help to understand the results.

**Fig. 2:**
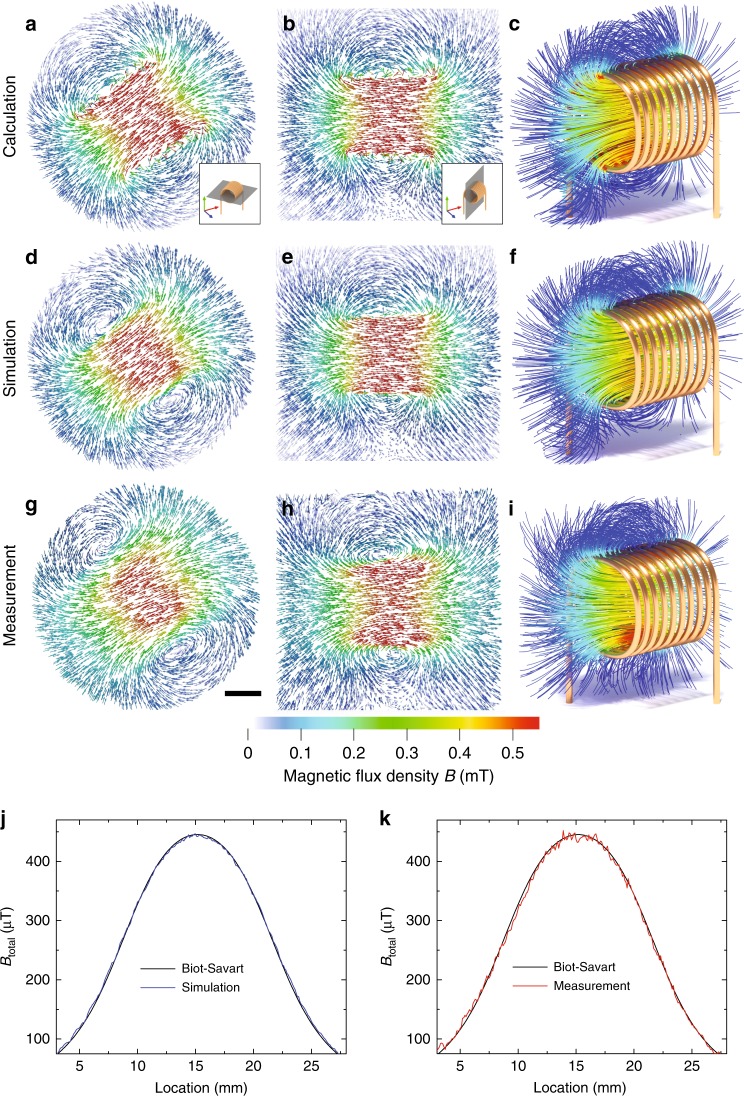
Vectors of the magnetic field produced by an electric coil (including leads). **a**–**c** As calculated by the Biot-Savart law, see Methods, Eq. (8). **d**–**f** TMART reconstruction of a simulated (ideal) measurement based on the theoretical field distribution of **a**–**c**. **g**–**i** Measured magnetic vector field distribution of the real coil. **a**, **d**, **g** Magnetic field vectors along the horizontal plane as indicated in the inset in **a** and **b**. **b**, **e**, **h** Magnetic field vectors along the vertical plane (see inset in **b**). **c**, **f**, **i** 3D visualization of selected magnetic field lines. Scale bar, 5 mm. **j** Magnetic flux distribution along the central symmetry axis of the electric coil. Calculated magnetic flux distribution (black line) and magnetic flux taken from the simulated measurement (blue line). **k** Same as **j**, but for the measured magnetic flux distribution

First, we prove the reliability of the TMART algorithm by reconstructing the well-known magnetic field of an electric coil that is calculated using the Biot-Savart law (Fig. 2a–c). Figure 1b and Supplementary Movie 1 show some selected magnetic field lines taken from the calculated magnetic vector field to demonstrate the complexity of the field. Based on the calculated magnetic vector field we simulated an ideal neutron tensor tomographic measurement and used the virtually created nine 3D data sets as an input for our algorithm. Figure 2d–f show the results.

In the second step, a real tensor tomographic measurement of an electrical coil of nearly the same dimensions and parameters as the simulated one is performed and reconstructed, leading to Fig. 2g–i. Despite some limits in spatial resolution the magnetic vector field is reconstructed very well. Quantitative comparison between calculated, simulated and measured magnetic flux distributions in Fig. 2j, k proves the validity of both the measurement and the algebraic reconstruction algorithm.

### Magnetic vector field inside a superconductor

The real strength of neutron tensor tomography lies in its applicability to bulk samples. We chose a cuboid-shaped sample, size 19.5 × 9.5 × 9.5 mm^3^, of polycrystalline lead, which is a weak neutron but strong X-ray absorber, to demonstrate the unique possibilities. Lead becomes superconductive below a critical temperature of 7.2 K. The sample is cooled down from room temperature to 4.3 K while applying a magnetic field of strength 0.5 mT (field cooling procedure, see Methods). Due to the Meissner effect most of the magnetic field is expelled from the interior of the superconductor. However some magnetic flux remains within the bulk, mainly in the (non-superconducting) grain boundaries of the polycrystalline material. After switching off the external magnetic field, the magnetic field trapped along the grain boundaries cannot easily vanish because a permanent electric current is induced in the surrounding superconducting grain according to Lenz’s law that then maintains the field.

**Fig. 3:**
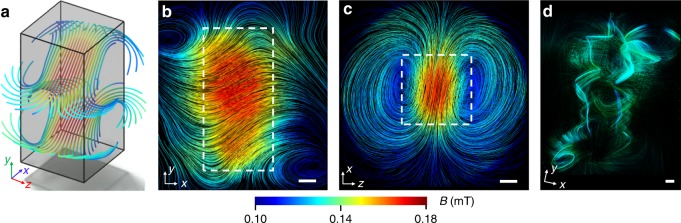
Magnetic vector field inside a superconducting lead sample measured at *T* = 4.3 K. **a** Some selected magnetic field lines show the location of the magnetic field inside the sample indicated by the cuboid. **b** Magnetic field lines in a selected *xy* plane (silhouette of the lead sample marked by dotted lines). Scale bar, 5 mm. **c** Magnetic field lines in a selected *xz* plane. Scale bar, 5 mm. **d** Selected swirling magnetic field lines as also seen in Supplementary Movie 2. Scale bar, 5 mm

TMART reconstruction of the measurement reveals the structure of the trapped magnetic vector field (Fig. 3a–c). It consists of a stronger trapped field inside the bulk with vortices outside. Figure 3d and Supplementary Movie 2 show selected magnetic field lines in and around the superconductor and illustrate the complexity of the magnetic field and the power of the measurement technique to deal with complex vortex-like magnetic field structures.

**Fig. 4:**
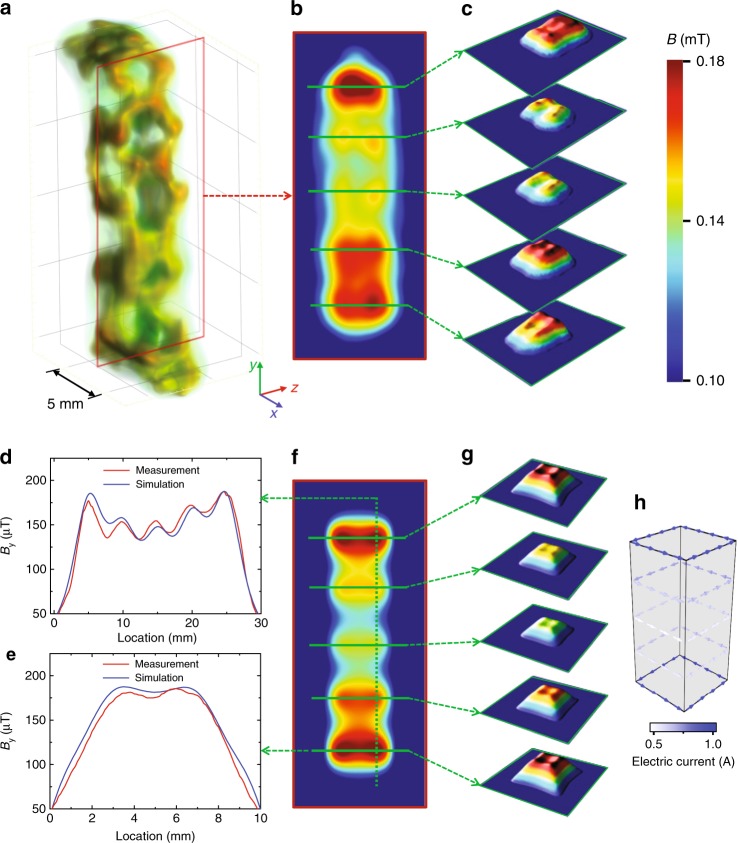
Three-dimensional distribution of the *y*-component of the magnetic vector field inside the bulk of the superconductor at 4.3 K as measured by tensorial neutron tomography. **a** 3D visualization of the magnetic flux density, where the red rectangle partially delimits the sample. The color scale used is non-linear as it is obtained by strong dynamic compression to visualize strong and weak fields at the same time. **b** Color-coded magnetic flux density (*y*-component) in the vertical cross section (*yz*-plane). **c** Magnetic flux distribution in selected horizontal planes as marked by the arrows. **d**, **e** Magnetic flux density distribution along the cross sections indicated in **e** for both measurement and simulation. **f**, **g** correspond to **b**, **c**, but show simulated magnetic flux distributions. **h** Schematic drawing of the calculated current flows in the superconductor

The measured magnetic flux density distribution inside the superconductor exhibits strong variations in intensity and field orientation in the region of trapped flux, Fig. 4a–c, and features a network or filament-like structure (Fig. 4a and Supplementary Movie 3). The magnetic field component along the cuboid axis (*y*) shows several pronounced maxima and minima, color-coded in Fig. 4b, c or given numerically in Fig. 4d, e along selected paths. At the locations of the five maxima, horizontal cross sections (*xz* plane) are taken (Fig. 4b, c). They reveal a magnetic field structure that consists of four individual maxima (Fig. 4c, e).

### Simulation and discussion

These observations suggest how currents might flow in the superconductor and lead to a simple model (see Methods) allowing us to reproduce some of the main features found in the measurement. Variations in the magnetic field along the (main) symmetry axis of the cuboid (Fig. 4b, f) are reproduced by assuming corresponding variations in the strength of the electric current flow in the 5 planes along the $\vec y$ axis shown in Fig. 4b, f. The calculated magnetic flux distribution is shown in Fig. 4f, g. The horizontal magnetic field distributions exhibiting four maxima, a typical feature of thin rectangular shaped electric current flows, are reproduced (compare Fig. 4c, g, see also Fig. 4e). As the simulation is based on uniformly distributed currents within the planes shown in Fig. 4b,f no further features with a lower symmetry are reproduced.

Note that it is not this simulation that proves the correctness of tensor tomography but the experiments and simulations performed on the coil.

The microscopic origins of the measured non-uniformities of trapped magnetic flux should be related to structural heterogeneities in the material. Since no flux is trapped within single grains due to the (complete) Meissner effect, the measured magnetic flux has to be assigned to the grain boundaries. Variations of the crystal grain size or impurities in the grain boundaries that act as pinning centers for the trapped flux could give rise to locally varying conditions. Supplementary Fig. 1 provides an image of the grain structure on the sample. Grain sizes range from a few 10 µm to some 100 µm all over the sample with strong local fluctuations. Areas containing smaller grains also contain more grain boundaries and therefore trap more magnetic flux. Therefore, the non-uniformities of flux distribution shown in Fig. 4 appear very plausible.

### Conclusions and outlook

In conclusion, tensorial neutron tomography of the three-dimensional spatial distribution of magnetic vectors in bulk materials is obtained by using a combination of two spin-polarizers, four spin flippers and a neutron imaging setup and applying a new iterative mathematical reconstruction algorithm (TMART). We demonstrate the accuracy of the algorithm by first reconstructing a known magnetic field, after which the very complex convoluted magnetic vector field of trapped flux in a Pb type I superconductor is measured.

Magnetic tensor tomography will have a wide field of applications in magnetism research. The technique is non-destructive and non-invasive and therefore well suited for in-situ investigations. Applications range from measurements on high-*T*_c_ superconductors where flux trapping and pinning effects or density distributions of Abrikosov vortices under varying external conditions can be analyzed to magnetic suprafroths and possibly also magnetic phase transitions and domain structures. A wide range of applications is expected in engineering sciences, where the technique can be used, for example, to analyze currents in electrical devices such as batteries and fuel cells, or to make visible magnetic structures in thin electrical steels for electric engines and high-efficiency transformers. Because neutrons have a high penetration depth in most elements the technique is not restricted to specific materials. The technique can be implemented at any modern neutron imaging instrument.

Further optimization is possible in many ways, for example by using more efficient ^3^He spin-polarizers or velocity selectors instead of solid-state polarisers. Spatial resolution could be notably improved by using setups for high-resolution neutron tomography that have been recently introduced or by using future neutron microscopes that are under development^26,27^. With such improved setups, spatial resolutions down to some µm should be achievable. Furthermore, in experiments where periodically repeating magnetic field and phase changes can be produced (e.g., relaxation effects in superconductors) time-resolved stroboscopic detection techniques especially at spallation sources^28^ offer new opportunities for investigating magnetic phenomena on a time-scale of ms or even µs^29^.

## Methods

### Interaction of neutrons with magnetic fields

Because of its spin and intrinsic magnetic moment a neutron moving through a magnetic field can only take two possible eigenstates, spin up |↑〉 and spin down |↓〉, with the two different Zeeman energies $\pm \mu {\vec{\mathrm B}}$, where *μ* = −9.66236 × 10^−27^ J ⋅ T^−1^ is the magnetic moment of the neutron^30,31^. The equation of motion of spin ${\vec{\mathrm S}}$ is described by

2 $$\frac{d}{{dt}}{\vec{\mathrm S}} = \gamma \left( {{\vec{\mathrm S}}\left( t \right) \times {\vec{\mathrm B}}\left( t \right)} \right),$$

where *γ* = –1.83247 × 10^−8^ rad·s^−1^·T^−1^ is the gyromagnetic ratio of the neutron.

The polarization ${\vec{\mathrm P}}$ of an ensemble of many polarized neutrons is described by the sum of all spin vectors ${\vec{\mathrm S}}$ divided by the number of neutrons. For large ensembles of neutrons, ${\vec{\mathrm P}}$ behaves exactly like a classical magnetic moment ${\vec{\mathrm \mu }}$ that experiences a torque $\vec \Gamma$ within a magnetic field^32^:

3 $$\vec \Gamma = {\vec{\mathrm \mu }} \times {\vec{\mathrm B}}{\mathrm{.}}$$

The magnetic moment $\vec \mu$ starts precessing with the Larmor frequency:

4 $$\omega = \gamma B.$$

After traveling for a certain time *t* and distance *x* through a magnetic field ${\vec{\mathrm B}}$ the neutron beam polarization ${\vec{\mathrm P}}$ undergoes a rotation $d\vartheta _{\vec B}$ around an axis parallel to ${\vec{\mathrm B}}$ of

5 $$d\vartheta _{\vec B} = \omega t = \omega \frac{x}{v} = \frac{{\gamma {\mathit \lambda} m}}{h}Bx,$$

where *v* is the velocity of the neutron, *λ* the de Broglie wavelength of the neutron and *m* *=* 1.6749 × 10^-27^ kg the neutron rest mass.

Equation (5) shows that from the measurement of the modulus and the rotation axis of $d\vartheta _{\vec B}$ the magnetic field strength and orientation, i.e., ${\vec{\mathrm B}}$, can be calculated.

Measurement of both the rotation angle and the rotation axis makes it necessary to perform more than just a single measurement as done previously^24^, where these two values could be separated only for uniform magnetic fields but not for general fields. In our new approach, 9 measurements are performed as described in the main text and in Fig. 1.

### Experimental setup

The measurements are performed at the CONRAD/V7 facility at Helmholtz Center Berlin for Materials and Energy (HZB)^33^. The instrument is located at the end of a curved neutron guide providing a spectrum of cold neutrons between 2 Å and 6 Å. The imaging setup used consists of a Li scintillator screen that converts neutrons into visible light that is collected by a CCD camera (Andor DW436N-BV with 2048 × 2048 pixels). The effective pixel size in all the measurements is 30 × 30 × 30 µm^3^. The spatial resolution of the set-up was about 200 µm (measured with a Siemens star test pattern). For the tensorial neutron tomography measurements a wavelength of 3 Å was chosen.

The solid-state spin-polarizers^34^ P_1_ and P_2_ in Fig. 1 consists of several 250-µm thick bent Si wafers coated on one side with polarizing FeCo supermirrors and on the other side with strongly neutron absorbing Gd and positioned in a magnetic field. These two coatings either deflect or absorb neutrons depending on their spin orientation relative to the magnetic field. The curvature causes a displacement of 250 µm halfway through the spin-polariser and thus avoids straight and undeflected beam paths. The measured transmission is approximately 30%, the beam cross-section 15 mm × 40 mm (width × height) and the measured degree of spin-polarization ∼95%.

A closed-cycle refrigerator with neutron-transparent aluminum windows is used for sample cooling down to 4.3 K. A cylindrical Helmholtz coil set-up with coil diameters of 600 mm generated the magnetic fields for the flux trapping experiments. A superconducting cuboid-shaped lead sample of dimensions 19.5 × 9.5 × 9.5 mm^3^ is cooled to 4.3 K while an external magnetic field of 0.5 mT (measured with a Hall probe) is applied (field cooling procedure). Then the external field is switched off and the tensorial neutron tomographic measurement procedure started.

### Measurement procedure

One exemplarily setting for the spin flippers is shown in Fig. 1a. Neutron beam spin polarization is flipped by $\frac{\pi }{2}$ around the $\vec z$ axis (the flight path of the neutrons as indicated by the vector arrows in Fig. 1a) in two steps by two separate $\frac{\pi }{2}$ flips, first around the $\vec x$ (by F_1_), then around the $\vec y$ axis (by F_2_) (i.e., the polarization will finally become parallel to the $\vec x$ axis). If no spin-polarization change is caused by the sample (as shown in Fig. 1a) the spin is flipped back into its original orientation around the $\vec z$ axis by the second pair of spin flippers (F_3_/F_4_). This flipper configuration is called $\vec x_i\vec x_f$ below. Therefore, the transmission signal through analyzer P_2_ (see Fig. 1a) is (ideally) 100% if one ignores the attenuation caused by neutron absorption or/and scattering by the sample (and the analyzers P_1_ and P_2_). Any modification of polarization by the sample will reduce transmission to a value below 100%.

In total there are 3×3 possible flipper configurations termed $\vec x_i\vec x_f$, $\vec x_i\vec y_f$, $\vec x_i\vec z_f$, $\vec y_i\vec x_f$, $\vec y_i\vec y_f$, $\vec y_i\vec z_f$, $\vec z_i\vec x_f$, $\vec z_i\vec y_f$, $\vec z_i\vec z_f$, where, e.g., $\vec x_i\vec z_f$ means that flippers F_1_/F_2_ initially rotate into the $\vec x$ axis, while F_3/_F_4_ finally flip into the $\vec z$ axis. All these configurations are applied in 9 independent measurements allowing us to measure the change in spin-polarization of the neutron beam along any path through the sample, thus yielding a radiograph of spin rotations. For a three-dimensional measurement of the magnetic vector field (tomography), the sample is rotated stepwise covering a full circle^35^. For each rotation step, nine radiographic projection images ($\vec x_i\vec x_f$ through $\vec z_i\vec z_f$) are taken. 300 such radiographic projection sets are acquired for each tomography with 120 s exposure time for each projection.

The maximum measurable magnetic field strength strongly depends on the extension and on the spatial resolution achieved. The smaller the extension of the field (and hence the sample) and the higher spatial resolution, the higher is also the possible magnetic field strength that can be measured. With the settings of the set-up presented here, arbitrary magnetic fields of some mT strength can be measured within a volume of some 10 mm^3^. The TMART algorithm (see below) allows for the incorporation of a priori assumptions. If a rough estimate of the magnetic field strength is available the values iterated by the algorithm can be limited to a certain range. In this case, stronger fields are accessible provided the variations of the field strength within the region of interest are not larger than 10 mT. In addition, a combination with high-resolution neutron imaging setups and possibly spin-echo or multi-wavelength techniques could further shift this limit to much higher magnetic field strengths (over 100 mT) in future experiments.

The lower limit for the measureable magnetic field strength depends very much on the quality of shielding of the magnetic field of the Earth and other external effects. In the measurement presented here on a superconductor the lower limit was in the range of µT.

### Tomographic reconstruction technique

The new tensorial algebraic reconstruction technique (TMART) is based on the known MART algorithm^36^, but uses 3 × 3 tensors instead of scalar values (see general overview in Supplementary Fig. 2). In contrast to MART, where only a single 3D data set is used as an input to create another single 3D data set as an output, the TMART algorithm is based on tensor calculations that calculate the three different components of the magnetic vector field from a set of nine spin-polarized neutron imaging measurements.

A neutron beam passing the volume element *α*_*i*_ within the sample will undergo a rotation around the magnetic vector field orientation unit vector $\vec n_i$. This rotation is described by the tensor $T\left( {\vec n_i,\alpha _i} \right)$ (a rotation matrix). The rotation angle has to be below 180°, which is ensured by choosing a small enough volume element or limiting the magnetic field. After moving through a series of volume elements $\alpha _1$, $\alpha _2$, …, $\alpha _N$ the corresponding tensor $T_{{\mathrm{final}}}\left( {\vec n,\alpha } \right)$that describes the resulting total rotation of neutron spin polarization is

6 $$T_{{\mathrm{final}}}\left( {\vec n,\alpha } \right) = T\left( {\vec n_N,\alpha _N} \right) \cdot \ldots \cdot T\left( {\vec n_i,\alpha _i} \right) \cdot \ldots \cdot T\left( {\vec n_1,\alpha _1} \right) = \mathop {\prod }\limits_{i = N}^1 T\left( {\vec n_i,\alpha _i} \right).$$

The neutron beam polarization after passing the sample $\vec P_{{\mathrm{final}}}$ (see main text) then is

7 $$\vec P_{{\mathrm{final}}} = T_{{\mathrm{final}}}\left( {\vec n,\alpha } \right) \cdot \vec P_0.$$

The TMART algorithm is an iterative algorithm that starts with constant values for the entire magnetic vector field. Then, a set of 9 projection images (i.e., a measurement of this magnetic vector field is simulated) and their deviations from the (real) measurement results are calculated. From this comparison, correction multipliers for the magnetic vector field, i.e., for the tensors used in Eq. (6), are calculated in order to partially correct the magnetic field values. With the new updated results for the magnetic vector field the next iteration steps starts. The strength of the multiplicative corrections is weighted with a relaxation (or damping) factor that is constantly reduced during iteration to prevent divergence of the procedure. A path length correction is implemented as the intersection of a given ray with a quadratic pixel may have a varying length, see Supplementary Fig. 3. MART is different and computationally more challenging than the filtered backprojection algorithm usually used for tomography but allows for solving the problem of tensorial tomography in an elegant way^37^.

### Simulation of the coil and the trapped magnetic flux

The magnetic vector field $\vec B\left( {\vec r} \right)$ generated by an electric current at location $\vec r$ is calculated by using the Biot-Savart law

8 $$\vec B\left( {\vec r} \right) = \frac{{{\mathrm{\mu }}_0}}{{4\pi }}\mathop {\scriptstyle\int} \limits_C^{} \frac{{I{\mathrm d}\vec l \times \vec r\prime }}{{\left| {\vec r\prime } \right|^3}},$$

where *I* is the electric current along segment ${\mathrm d}\vec l$ of path *C* and $\vec r\prime$ the distance between ${\mathrm d}\vec l$ and $\vec r$. The electric coil has an inner diameter of 10 mm, a length of 12 mm and 9.5 turns and is made of 1-mm thick aluminum wire. The electric current applied is 0.75 A. Due to limitations of manufacture the geometry of the measured coil slightly differs from the ideal shape assumed for the calculation and simulated measurement.

For calculating the field in the superconductor, electric currents circulating around the superconductor in the five planes marked by horizontal green lines in Fig. 4f are assumed. More specifically, current flow is along a rectangular path around the sample and equally distributed in a zone reaching from the outer edge of the superconductor to 2.5 mm below its surface. The five currents are varied in a way that the resulting magnetic flux fits to the measured results shown in Fig. 4a–c. From top to bottom in Fig. 4, the values are 0.933A, 0.655A, 0.535A, 0.584A, and 0.937A.

These calculations represent a simplification of the real current flows inside the superconducting sample that is in fact much less uniform and distributed over the whole volume as can be clearly seen in Fig. 4a–c and the Supplementary Movie 3.

## Electronic supplementary material

Supplementary Information

Description of Additional Supplementary Files

Supplementary Movie 1

Supplementary Movie 2

Supplementary Movie 3

## Data Availability

The data that support the findings of this study are available from the corresponding author upon reasonable request.
