# Supplementary material for: Tensorial neutron tomography of three-dimensional magnetic vector fields in bulk materials
Source: Nat Commun. 2018 Oct 2;9:4023. doi: 10.1038/s41467-018-06593-4 (PMC6168513; doi:10.1038/s41467-018-06593-4)
Supplement: Supplementary file 1 — Supplementary Information [file 41467_2018_6593_MOESM1_ESM.pdf]

Supplementary Information to

Tensorial Neutron Tomography of Three-Dimensional Magnetic Vector Fields in Bulk Materials

A. Hilger[1,2], I. Manke[1], N. Kardjilov[1], M. Osenberg[2], H. Markötter[1], J. Banhart[1,2]

[1]Helmholtz Centre Berlin for Materials and Energy (HZB), Institute of Applied Materials, Hahn-Meitner-Platz 1, 14109 Berlin, Germany

[2]Technische Universität Berlin, Department of Materials Science and Technology, Hardenbergstraße 36, 10623 Berlin, Germany

Supplementary Figures

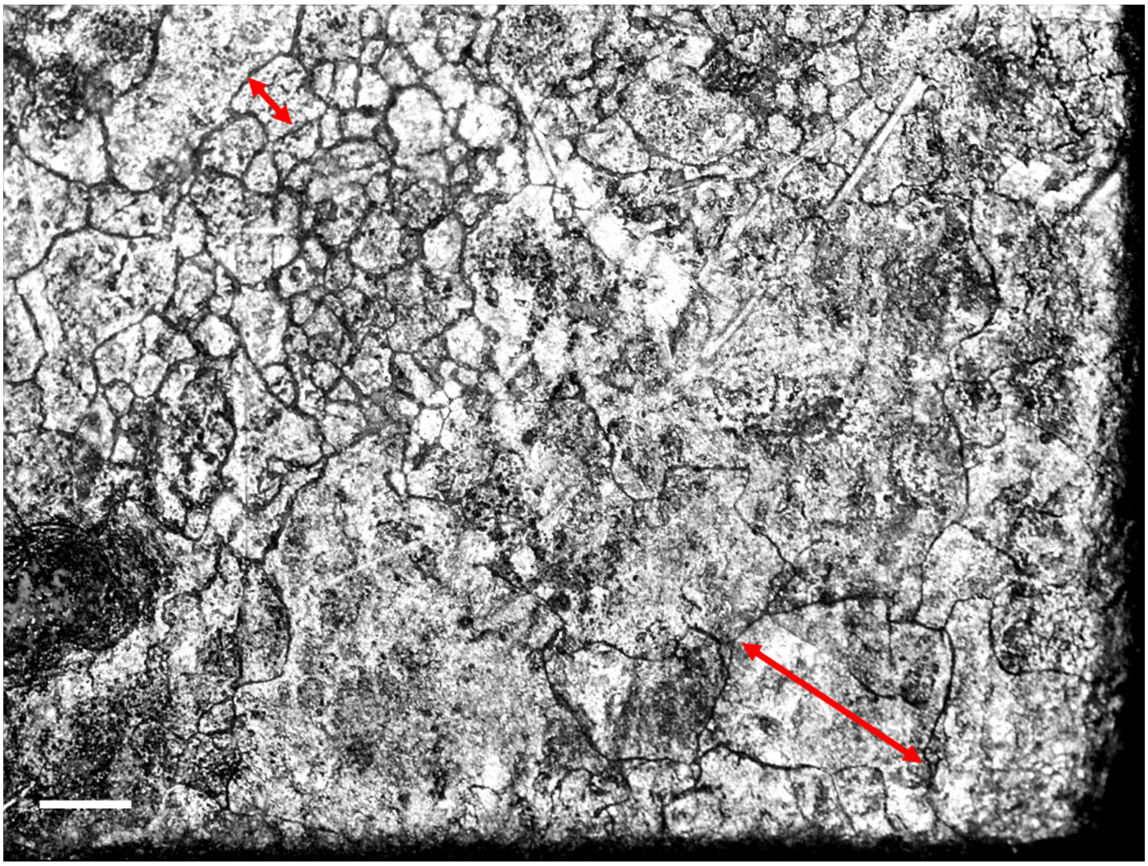

Supplementary Figure 1. Light microscopy image of the top surface of the investigated lead sample close to one of the edges (lower right). Grain sizes at different location vary strongly as indicated by the two arrows. Scale bar (white), 100 µm.

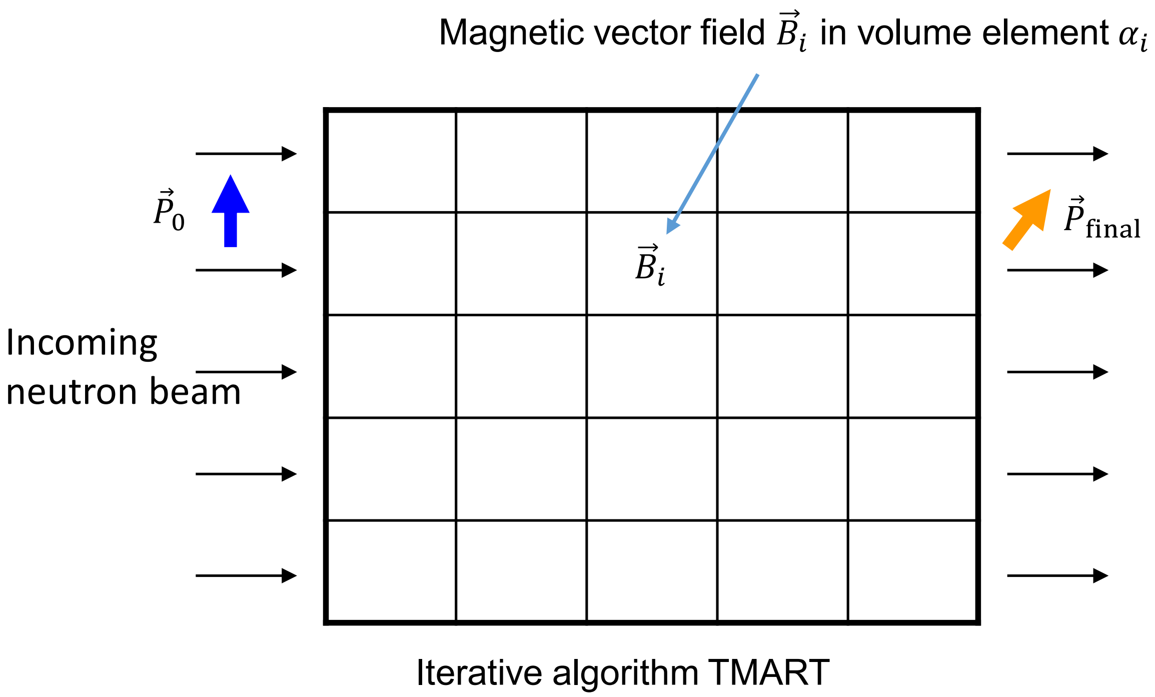

Supplementary Figure 2. Schematic drawing explaining the general measurement and iterative magnetic field reconstruction procedure (see text).

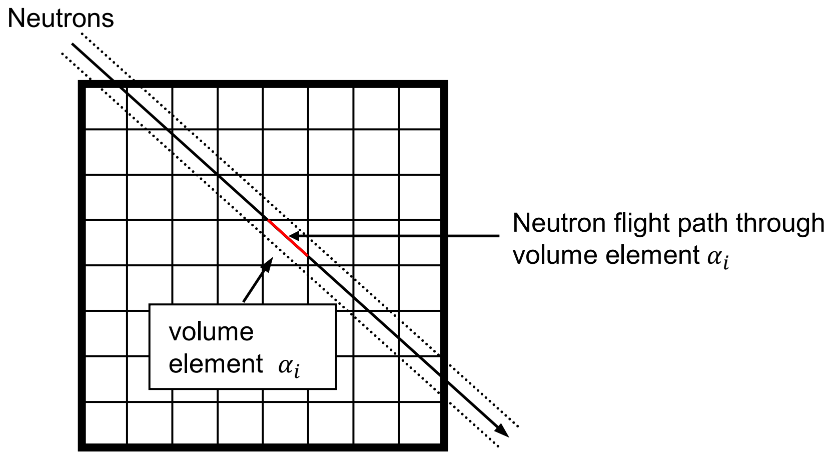

Supplementary Figure 3. Length correction for the TMART algorithm (see text). Three different possible neutron paths through volume element α_i are shown. The time the neutron stays in α_i differs for all three paths.
