# Supplementary material for: Tensorial neutron tomography of three-dimensional magnetic vector fields in bulk materials
Source: Nat Commun. 2018 Oct 2;9:4023. doi: 10.1038/s41467-018-06593-4 (PMC6168513; doi:10.1038/s41467-018-06593-4)
Supplement: Supplementary file 2 — Description of Additional Supplementary Files [file 41467_2018_6593_MOESM2_ESM.pdf]

Description of Additional Supplementary Files

File Name: Supplementary Movie 1
Description: Selected magnetic field lines around an electric coil (calculation, see text, methods section and Fig. 1)

File Name: Supplementary Movie 2
Description: Selected swirling magnetic field lines in and around a superconducting lead sample held at 4.3 K. Fig. 3d represents one special view.

File Name: Supplementary Movie 3
Description: 3D visualization of the magnetic flux density in a superconducting lead sample at 4.3 K (Note: The applied colour scale used is non-linear as it is obtained by strong dynamic compression to visualise strong and weak fields at the same time).
